# Cardiac magnetic resonance in systemic sclerosis: imaging features and potential prognostic implications. A literature review

**DOI:** 10.3389/fmed.2025.1606593

**Published:** 2025-08-15

**Authors:** Giovanni Vitale, Matteo Colina, Domenico Attinà, Fabio Niro, Paolo Ortolani

**Affiliations:** ^1^Cardiology Unit, Ospedale Santa Maria della Scaletta, Imola, Italy; ^2^Service of Rheumatology, Section of Internal Medicine, Department of Medicine and Oncology, Ospedale Santa Maria della Scaletta, Imola, Italy; ^3^Department of Pediatric and Adult Cardio-Thoracovascular, Oncohematologic and Emergencies Radiology Unit, IRCCS Azienda Ospedaliero-Universitaria di Bologna, Bologna, Italy

**Keywords:** cardiac magnetic resonance, fibrosis, systemic sclerosis, pulmonary arterial hypertension, prognostic factors, myocardial inflammation

## Abstract

Systemic sclerosis (SSc) is a chronic, multisystem disorder characterized by vascular dysfunction, immune dysregulation with production of autoantibodies, fibroblasts dysfunction and consequent abnormal collagen production, leading to progressive fibrosis of the skin and various organs. Cardiac involvement is common, affecting the myocardium, pericardium, valvular structures and conduction tissue, even though it is often unrecognized. Despite this, it is a major determinant of morbidity and mortality in SSc, being responsible for about 15% of all deaths. Due to the relevant prognostic implications of cardiac involvement its early detection is mandatory. A comprehensive screening through a multimodality approach is required in all patients with SSc, even in those without overt cardiac symptoms. Cardiac magnetic resonance (CMR) is now considered the gold standard for non-invasive detection of the myocardial disease SSc related. It provides not only a morphological and functional assessment, but also offers an ultrastructural definition of the myocardium, particularly by the detection of fibrosis and myocardial inflammation (MI), unmasking an initial myocardial involvement since the early stage of disease. The aim of this review is to describe the potential spectrum of cardiac involvement in SSc, and to highlight central role of CMR in its detection, offering a comprehensive description of the imaging features and their prognostic implication.

## 1 Introduction

Systemic sclerosis (SSc) is a generalised autoimmune disorder of connective tissue affecting skin and internal organs. The mechanisms involved in the pathophysiology of the disease are not yet fully understood; fibrosis and microvascular occlusion characterise the pathologic findings seen in all involved organs (1). The clinical presentation can be pleomorphic, according to the organs involved, with several subsets described: limited cutaneous SSc (lcSSc), diffuse cutaneous SSc (dcSSC), and SSc *sine scleroderma*. Although SSc in an uncommon disease, it is important because it represents a paradigm for other more common medical conditions in which immunologically triggered fibrosis occurs, such as liver fibrosis and idiopathic pulmonary fibrosis. In managing patients with SSc, it is essential to routinely search for negative prognostic factors (2). Among these, cardiac involvement has a significant impact on overall survival. The heart is commonly affected, with involvement seen in over 70% of patients, and up to 80% in autopsy studies (3, 4). Cardiac involvement is responsible for the 20–30% of unexpected deaths in SSc (5, 6). However, it is often silent, remaining unrecognized until the late stages of the disease (7). All cardiac tissues—including myocardium, pericardium, valvular structures, coronaries and conduction system—can be affected. Cardiac damage may be primary, resulting from direct vascular, fibrotic and inflammatory injury to cardiac tissue, or secondary, as a consequence of other organs disease, such as pulmonary arterial hypertension (PAH), interstitial lung disease, or scleroderma renal crisis. Ischaemic injury due to small vessel vasculopathy and MI leads to myocytes necrosis, reperfusion damage, and ultimately to myocardial fibrosis. Microvascular disease affects the media and intima layers of the small arteries and arterioles, and, in combination with coronary vasospasm—often exacerbated by cold exposure—contributes to anginal chest pain and acute coronary syndromes. Additionally, microvascular disease is a key factor in the pathogenesis of PAH. Compared to idiopathic PAH, overall survival is lower in PAH associated with SSc, despite similar hemodynamic features (8). Chronic ischaemic injury, chronic MI and consequent progressive myocardial fibrosis lead to adverse myocardial remodelling and altered ventricular compliance, resulting in both diastolic and systolic dysfunction.

## 2 Cardiac magnetic resonance in systemic sclerosis

Although clinical evaluation, electrocardiogram and echocardiography still represent today the first approach to assess cardiac involvement in patients with SSc, their sensitivity and specificity are relatively low, so a large number of affected patients may remain undiagnosed. The advent of cardiac magnetic resonance (CMR) has significantly impacted the epidemiology and clinical management of various diseases, including SSc. CMR, a non-radiating imaging technique, is now regarded as the gold standard for non-invasive evaluation of cardiac morphology and function. It is more accurate and reproducible than echocardiography, and offers the possibility of tissue characterization of the myocardium, particularly in terms of fibrosis and MI, providing crucial insights for clinical management. The high sensitivity of CMR enables early detection of myocardial damage, even in its preclinical stage, in particular during initial inflammatory phase, before than fibrosis or overt functional and morphological changes occur. Furthermore, its non-radiating nature ensures patient safety, making it suitable for longitudinal monitoring during follow-up. The limited availability, the costs and the potential (few) contraindications are the main limitations.

### 2.1 Morpho-functional evaluation and diastolic function

Myocardial involvement in SSc can present with focal hypo- or akinesia, ranging to various degrees of global left ventricular (LV) systolic dysfunction and dilatation. Myocarditis and fibrotic replacement are responsible for segmental and/or global ventricular abnormalities in the acute and chronic phases, respectively. Right ventricular (RV) hypertrophy, dilatation and dysfunction—often associated with anomalous movement of the interventricular septum—suggest the presence of PAH. These findings, particularly when they are nuanced, can be misdiagnosed by echocardiography, whereas they are more easily to detect with CMR. Specific cardiac structures, such as the right ventricle, LV apex and atrial chambers, may be challenging to approach with ultrasound. CMR overcomes the limitations of the transthoracic echocardiography, by combining different cine images, even also in not canonical cut planes, providing a more comprehensive evaluation. Recently, five distinct CMR phenotypes of cardiac SSc have been described, based on the presence of dysfunction and/or dilatation of one or both ventricular chambers: dilated right heart with RV failure, biventricular dilatation and dysfunction, normal function with large cavity sizes, normal function with normal cavity, normal function with small cavity, the latter two subsets being associated with a more favourable outcome (9). Several studies evaluated the potential correlation between morpho-functional parameters and clinical outcomes, often with conflicting results (10). Among these parameters, RV ejection fraction has been shown to independently predict all-cause mortality (9). SSc patients frequently develop signs and symptoms of heart failure (HF), regardless of systolic function. According to the results from the European Scleroderma Trials and Research (EUSTAR) registry, 36.2% of SSc patients with cardiac involvement meets diagnostic criteria for HF with preserved ejection fraction (HFpEF), while HF with reduced (HFrEF) or mildly reduced ejection fraction (HFmrEF) has been reported much less frequently, each accounting for 1.5% of cases (11). Indeed, LV systolic dysfunction has been reported in about 5.4% of cases; however, MI and fibrosis can alter biventricular myocardial relaxation and compliance, leading to elevated pulmonary venous pressures, thereby increasing RV afterload. This results in RV hypertrophy and subsequent diastolic dysfunction (DD), characterized by reduced RV filling time and prolonged isovolumic relaxation time, the latter indicating impaired active myocardial relaxation (12). As RV DD progresses, maladaptive remodeling with chamber dilatation develops, ultimately leading to RV systolic dysfunction. This, in turn, adversely affects left-sided chambers, further compromising LV filling. DD is closely associated with HFpEF, and numerous studies have evaluated it non-invasively using echocardiography. Tennøe et al. reported that 17% of 275 consecutive SSc patients had DD at baseline, with the prevalence increasing to 29% after a 3.4-year follow-up. Patients with DD were older and had higher rates of systemic hypertension, ischemic heart disease, atrial fibrillation, and pulmonary hypertension compared to those without DD. The presence of DD was associated with poorer survival (13). Accordingly, diastole should systematically be evaluated, being often the only functional detectable abnormality. While echocardiography remains the traditional approach for its non-invasive assessment, several applications of CMR imaging have demonstrated potential utility in the evaluation of both LV and RV diastolic function, including cine imaging for ventricular filling dynamics, phase-contrast sequences for transvalvular flow analysis, feature tracking for diastolic strain rates, and T1 mapping to detect myocardial fibrosis and impaired compliance, although the evidences remain limited (14–16).

### 2.2 Tissue characterization

MI, resulting from aberrant activation of the immune system, is common in SSc, although it is often clinically silent. Overt clinical myocarditis is rare, whereas recent CMR studies have highlighted the critical role of subclinical inflammation in the pathogenesis of cardiac damage. Since 2009, the definition of the Lake Louis criteria has provided the foundation for non-invasive diagnosis of myocarditis (i.e., MI), based on the identification of three diagnostic criteria: edema, hyperemia, and necrosis. These criteria are derived from T2-weighted, early gadolinium enhancement, and late gadolinium enhancement (LGE) CMR images, respectively: for the diagnosis of myocarditis, at least two of the three criteria must be present (17, 18). The advent of CMR mapping imaging has advanced tissue characterization, improving sensitivity and specificity through direct measurement of T1 and T2 relaxation times. As a result, the Lake Louis diagnostic criteria have been revised in 2018 (19). Accordingly, both myocardial edema (described as regional or global increase of native T2 or T2 signal intensity) and non-ischaemic myocardial injury (i.e., regional or global increase of native T1 or extracellular volume (EVC), or presence of LGE) must be present. The coexistence of pericardial involvement and/or LV systolic—global or regional—dysfunction further support the diagnosis.

#### 2.2.1 Edema and hyperemia

Immune dysregulation in SSc leads to the upregulation of endothelial adhesion molecules, leukocytes diapedesis and release of cytokines, with consequent increase in tissue free water content. On CMR analysis, edema prolongs both T1 and, particularly, T2 relaxation times. In T2-weighted short-tau inversion recovery (STIR) images, edema appears as regional or global signal hyperintensity. However, poor image quality and limited sensitivity can hinder the detection of diffuse and subtle edema, which is typical of SSc, especially when muscle signal intensity cannot be used as reference due to coexistent skeletal myositis. The introduction of cardiac T1 and T2 mapping has helped overcome the limitations of conventional CMR. Compared to T2-wheighted imaging, both T1 and T2 mapping techniques have shown greater sensitivity for the detection of MI. In particular, T2 mapping can directly measure the prolongation of myocardial T2 relaxation time, identifying free water related to MI or acute ischemia with more accuracy than traditional T2-weighted imaging (20, 21). Myocardial T1 is also influenced by edema, even though the increase in native T1 is less specific, since it reflects both the intracellular and extracellular space, and is also influenced by fibrosis and capillary leak (19). The addition of T1 and T2 mapping improves the diagnostic yield of CMR in detecting MI, increasing sensitivity from 52 to 89%, compared to traditional CMR (22). Furthermore, hyperemia and increased vascular permeability can be detected using early gadolinium enhancement, which manifests as an increased signal in T1-weighted post-contrast images due to the interstitial uptake of the contrast agent. Elevated native T1 and/or T2 has been found in over 62% of SSc patients with normal conventional CMR (i.e., negative LGE and T2-weighted images), highlighting that subclinical myocardial damage is common, even in patients without overt heart disease (23). The detection of an early myocardial inflammatory stage provides the opportunity for anti-inflammatory therapies, preventing the progression towards irreversible fibrosis (24).

#### 2.2.2 Fibrosis

**Figure 1:**
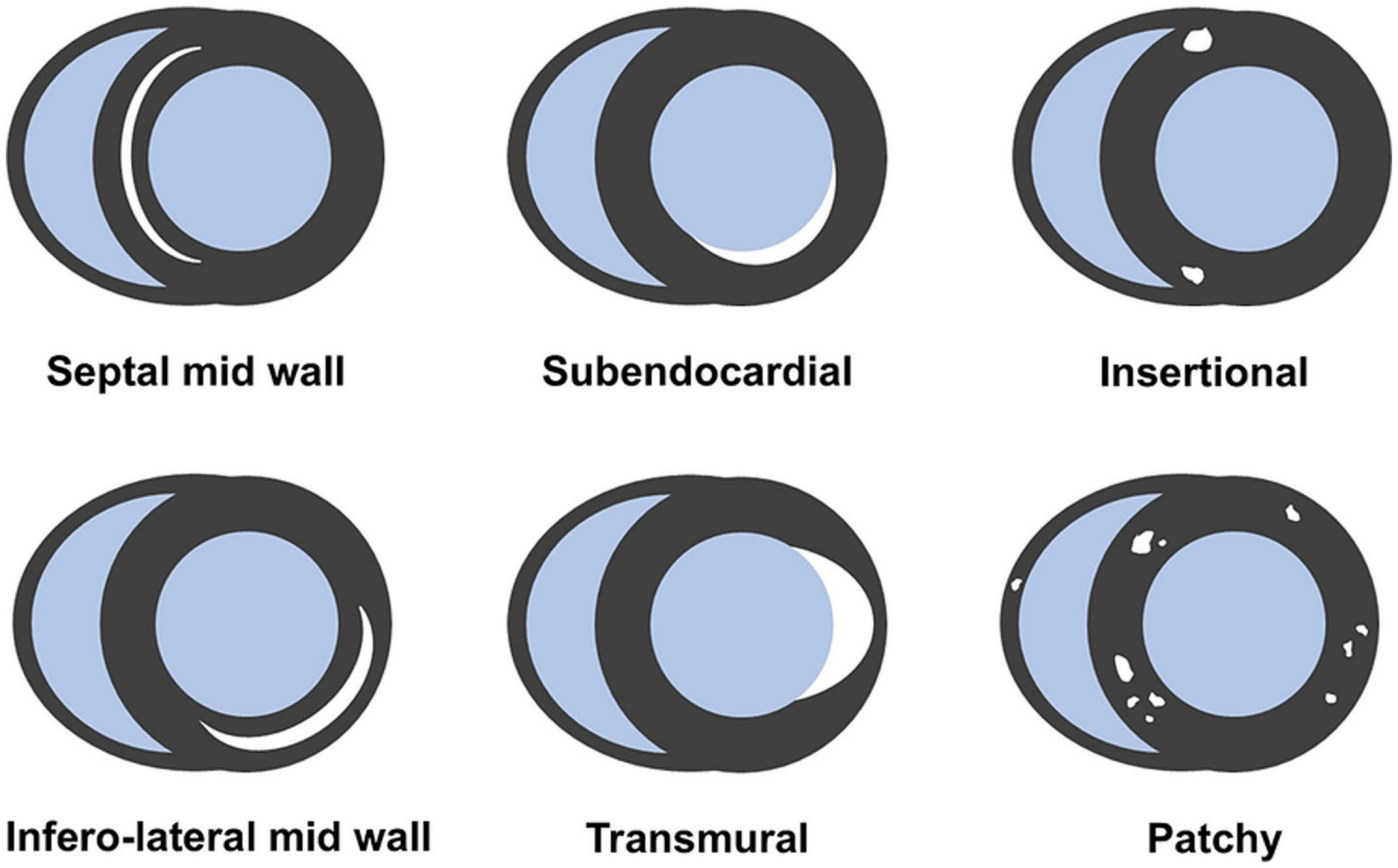
Different patterns of late gadolinium enhancement in systemic sclerosis.

**Figure 2:**
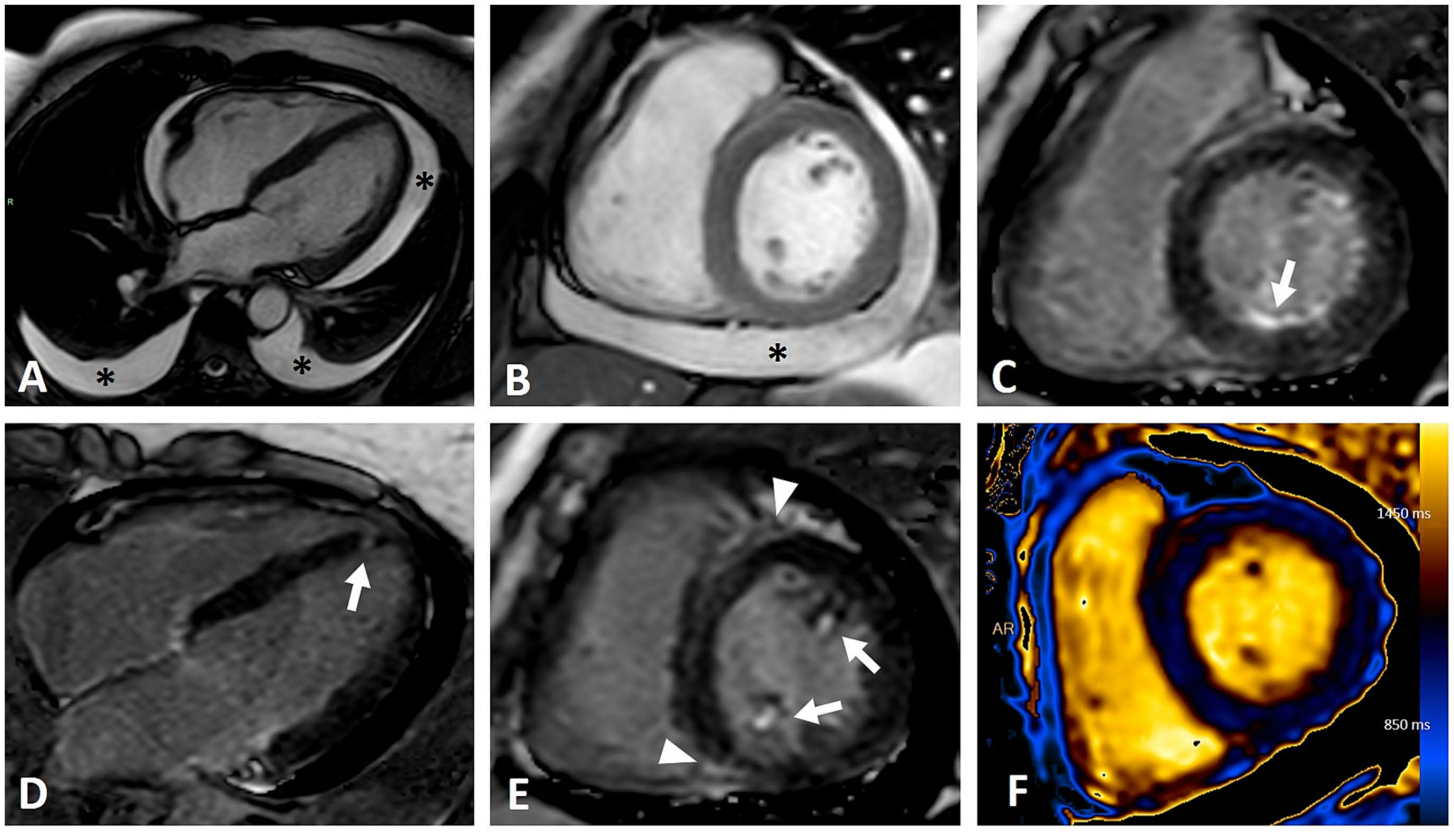
CMR imaging of a female patient with diffuse cutaneous systemic sclerosis and heterogeneous cardiac involvement. **(A,B)** Moderate pericardial and bilateral pleural effusion (asterisks) in four-chambers **(A)** and short axis **(B)** steady-state free precession (SSFP) sequences. **(C–E)** Inversion recovery sequences demonstrating various patterns of LGE: infero-basal subendocardial (**C**, arrow), focal septal apical (**D**, arrow), papillary muscles involvement (**E**, arrows), insertional (**E**, arrowhead). **(F)** T1 mapping showing abnormal elevated values (mean value of 1,100 ms) due to myocardial fibrosis and edema.

Myocardial fibrosis is the pathogenic hallmark of cardiac involvement in SSc, and has been reported in over 80% of cases in autoptic studies (3, 25). CMR enables non-invasive detection of myocytes loss and myocardial fibrosis, and is now considered the gold standard for this purpose. The presence of LGE significantly influences overall outcome, so its systematic research is mandatory (10, 26). Various CMR studies have reported a wide range of LGE prevalence in SSc, from 15 to over 60% (27). Several distinct patterns of LGE have been described (Figure 1): mid wall linear distribution is the most frequent, typically affecting the septal, infero-lateral, lateral or inferior mid/basal segments, following a non-coronary distribution (27, 28). Patchy and insertional LGE have also been reported, with the latter likely reflecting RV overload due to PAH; ultimately, mixed pattern and subendocardial to transmural LGE have been observed (Figure 2) (29, 30). The RV free wall is also affected by replacement fibrosis; however, its detection is challenging and may be underestimated due to the peculiar characteristics of the RV wall (thin and trabeculated). The presence of myocardial fibrosis is associated with a lower LV ejection fraction, and a relevant amount of LGE has been found in patients with ventricular arrhythmias (27, 29, 30). Prognosis is significantly worsened by the presence of LGE, with an event-free 5-year survival rate of less than 50% in such cases (26). While LGE can identify replacement fibrosis, interstitial fibrosis is more diffuse and nuanced, and may be missed by traditional post-contrast imaging. More recent parametric mapping techniques, particularly native T1 and extracellular volume (ECV), provide an accurate assessment of tissue relaxation times, enhancing diagnostic sensitivity for detecting diffuse fibrosis rather than focal scarring. Native (pre-contrast) T1 relaxation time is influenced by changes in both extracellular and intracellular spaces, occurring in conditions where free water is present, such as edema, as well as in fibrosis or amyloidosis, where water is bound to large molecules like collagen (31, 32). Elevated native T1 is detectable in more than half of SSc patients, representing the only abnormal parameter in approximately one-third of cases (33). Myocardial ECV is indirectly measured from the ratio of T1 changes before and after contrast administration. ECV is a precise indicator of the myocardial extracellular space, and proves particularly useful in infiltrative diseases like cardiac amyloidosis, where the interstitial space is abnormally high. Accordingly, native T1 and ECV are sensitive surrogates of diffuse, interstitial fibrosis, and appear to be significantly elevated in SSc patients compared to healthy controls. Both T1 and ECV correlate with N-Terminal-pro-Brain Natriuretic Peptide levels, disease severity and activity, and predict adverse outcomes in SSc patients (18, 34). Gotschy et al. found no significant differences in LV function, volume, or LGE in early stage SSc patients with high and low native T1, suggesting that that conventional CMR may not effectively identify patients at increased risk (18). Bordonaro et al. reported that elevated T1 and ECV are independent predictors of adverse events (including cardiac death, haemodynamically significant arrhythmia, or heart failure) in SSc patients (34). These findings highlight the additional contribution of CMR mapping to risk stratification compared to standard LGE, and suggest that it should be systematically evaluated in patients with SSc.

### 2.3 Deformation imaging

Traditional echocardiographic parameters are often ineffective for detecting subclinical systolic impairment. However, reduced LV and RV global longitudinal strain (GLS) is common among patients with SSc and has been associated with an increased risk of all-causes mortality and hospitalization (35–37). A basal–apical gradient has also been observed, with basal segments being more affected than the apex in both ventricles (35, 38). More recently, feature-tracking CMR analysis has emerged as a novel method for studying biventricular deformation and, accordingly, myocardial performance. Several studies have evaluated LV deformation in SSc using future-tracking analysis, showing that LV and RV strains are often impaired despite preserved LV ejection fraction (39, 40). Gotschy et al. reported the potential prognostic utility of deformation analysis, demonstrating that reduced GLS and elevated T1 can identify subjects at increased risk of death for any cause (18). Faher et al. demonstrated that GLS assessed by feature-tracking predicts overall survival regardless of cardiac output, reduced LVEF, and LGE (41). Moreover, some studies have reported significant improvement in LV and RV strain after the initiation of specific therapies for PAH, suggesting the potential utility of strain imaging for monitoring the response to therapies (42). However, data are still scarce, and further evidence is warranted.

### 2.4 Pulmonary arterial hypertension

PAH is common in SSc, with an estimated prevalence of 7–12% (43). Compared to idiopathic one, prognosis in PAH related to SSc is worse. One potential explanation seems to be a more impaired RV pump function, likely due to intrinsic abnormal collagen deposition. Additionally, microvascular disease and resulting chronic ischaemic damage have been postulated as contributing factors (44). Although CMR cannot directly measure pulmonary pressures, it provides valuable information on RV wall thickness, volumes and function, complementing echocardiographic assessment. Furthermore, assessment of fibrosis, measurement of myocardial strain and precise quantification of pulmonary and tricuspid regurgitant volumes are other potential applications. Patients with SSc-associated PAH have higher native myocardial, T2 and ECV, compared with patients without, and have more frequently pericardial effusion (9). Insertional myocardial scar, detected by LGE or native T1 elevation, is indicative of RV pressure overload, and correlates with disease severity (45). More recently, Knight et coll. Demonstrated that native T1 and indexed RV end-systolic volume are independent predictors of all-cause mortality in SSc-associated PAH, providing also potential thresholds to identify patients with a poorer prognosis (46). Given the growing body of evidence, CMR has become an integral part for comprehensive risk assessment in PAH together with the traditional prognostic predictors, offering the advantage of being non-invasive and reproducible over time.

### 2.5 Pericardial involvement

The pericardium is frequently affected, with involvement ranging from 33 to 72% (47). Asymptomatic mild pericardial effusion is the most common finding, whereas clinically symptomatic pericardial disease is less frequent. Some patients may present with typical acute pericarditis, characterized by elevated serum inflammatory biomarkers, chest pain and pericardial rubs. Constrictive pericarditis and pericardial tamponade, although possible, occur infrequently. CMR is typically considered a second-line imaging modality for diagnosing acute pericarditis; however, its high resolution and ability to perform tissue characterization make CMR the gold standard for non-invasive evaluation. A thickened pericardium of ≥ 3 mm can be seen in both acute and chronic pericarditis. Cine imaging offers a comprehensive functional assessment of the cardiac chambers and pericardial space, revealing pericardial effusion and its potential effects on cardiac functions, such as right chambers collapse in cardiac tamponade, or septal bounce in constrictive pericarditis at real-time cine images.

### 2.6 Valvular heart diseases

Heart valves are also affected by the immune dysregulation of SSc. Nodular thickening of the tricuspid, aortic and especially mitral valves (in approximately 38% in autoptic studies), along with retraction of the chordae tendineae and consequent valvular regurgitation, are the most common valvular alteration observed (48, 49). Also anecdotical cases of non-bacterial thrombotic endocarditis have been reported (50). Due to its superior temporal resolution and widespread availability, echocardiography remains the first approach for evaluating heart valves. However, CMR, using phase contrast imaging, can be a valuable alternative for approaching valvular disorders, especially in cases with poor acoustic windows, or when results are inconclusive, providing a more precise quantification of valvular regurgitation.

### 2.7 Microvascular dysfunction

Myocardial damage in SSc is also caused by repeated chronic ischaemic injury, resulting from structural microvascular impairment and abnormal vasoreactivity, rather than epicardial coronary arteries disease. Advances in myocardial perfusion imaging have spurred growing interest in the evaluation of microvascular dysfunction in SSc. The first demonstration of cold-induced coronary vasospasm in SSc was documented in several nuclear medicine studies, who found reversible myocardial perfusion defects after cold exposure, that seem to predict cardiac events and mortality in SSc (51–53). More recently, even CMR has found application in this context. Inducible subendocardial perfusion defects have been reported in about 79% of the patients assessed by stress CMR with adenosine, and resulted to be associated with higher plasmatic levels of ultrasensitive C reactive protein, suggesting a potential link between chronic MI and microvascular dysfunction (30). Gyllenhammar et al. demonstrated that patients with SSc exhibit decreased myocardial perfusion during adenosine stress, but not at rest, compared to healthy controls (54). Furthermore, Galea et coll. Reported a reduced vasodilatory response to the cold pressure test in SSc patients without cardiac symptoms, indicating a potential early role of endothelial microvascular dysfunction in the pathogenesis of cardiac damage (55). However, evidences regarding the potential prognostic implications of microvascular impairment remain limited and warrants further investigation.

## 3 Conclusion

Cardiac involvement in SSc is frequent, often subclinical, and carries an ominous prognosis. Given its relevant prognostic implications, early detection is mandatory, in order to timely start specific therapies and potentially prevent irreversible damage. Growing evidence underscores the central role of CMR in identifying subclinical cardiac involvement in SSc. CMR provides a non-invasive, multiparametric assessment through precise morphological and functional evaluation. Tissue characterization enables preclinical detection, even without the use of contrast agent, especially with recent advancements in mapping techniques. This capability can help clinicians to better understand the complex pathogenesis of cardiac damage. Furthermore, thanks to its non-radiating nature, CMR permits to safely monitor SSc patients at follow-up. Unfortunately, the well acknowledged utility of CMR is limited in real-world practice due to its elevated costs, scarce availability, and the prolonged time for both acquisition and analysis process. Further evidences are still need to elucidate the potential role of CMR in predicting outcomes and monitoring therapy response.
